# Anlotinib combined with radiotherapy and chemotherapy for recurrent pelvic osteosarcoma treatment: a case report and literature review

**DOI:** 10.3389/fonc.2023.1283932

**Published:** 2023-12-07

**Authors:** Qian Chen, Kai Zheng, Ming Xu, Ning Yan, Gong Hai, Xiuchun Yu

**Affiliations:** ^1^ Department of Orthopedics, The 960th Hospital of the People’s Liberation Army, Jinan, Shandong, China; ^2^ Department of Radiotherapy, The 960th Hospital of the People’s Liberation Army, Jinan, Shandong, China

**Keywords:** recurrent pelvis osteosarcoma, anlotinib, radiotherapy, adjuvant chemotherapy, case report

## Abstract

At present, the treatment of recurrent pelvic osteosarcoma is too simple, and most of the patients are treated with chemotherapy, radiotherapy, and/or combined surgery. Here, we report a 29-year-old man diagnosed with local recurrent pelvic osteosarcoma. Imaging showed that the tumor had obvious enhancement and abundant blood vessels. There was no indication of surgery. After the patient’s consent was obtained, we used anlotinib as a sequential treatment to chemotherapy. During the first course of adjuvant chemotherapy, we treated the patient with intensity-modulated radiotherapy (IMRT) with a total dose of 60 Gy equivalents. No disease recurrence was reported at 25 months after multimodal combination therapy.

## Introduction

Osteosarcoma is the most common primary sarcoma of the bone (1). It is typically treated with a combination of chemotherapy and wide resection. At present, the 5-year osteosarcoma-associated survival rate is approximately 70% (2). However, pelvic osteosarcoma, accounting for <10% of all osteosarcomas, has an expected 5-year survival rate of 18%–38% (3). In pelvic osteosarcoma, the tumor is commonly located in the posterior portion of the iliac wing and expands into the sacrum and the lower lumbar vertebrae. Because of slow growth, the initially noted mild to moderate pain is often mistaken for low back pain. As time passes, the tumor usually becomes huge (diameter > 10 cm) at initial diagnosis, which is a challenge for the local treatment of pelvic osteosarcoma. An R0 resection for pelvic osteosarcoma is less possible than that expected for extremity osteosarcoma (4, 5). This leads to a local recurrence rate of 11%–44% (6). For a long time, hindquarter amputation has been the primary treatment for recurrent pelvic osteosarcoma (4).

In recent years, as a new means of tumor treatment, targeted therapy has shown breakthrough results in the treatment of many types of tumors. Anlotinib became the first Level I recommendation to be included in the Chinese Society of Clinical Oncology (CSCO) guidelines as a second-line targeted treatment for advanced or unresectable bone and soft tissue tumors. In addition, some current basic studies and clinical reports also suggest that anlotinib shows certain potential in the treatment of advanced osteosarcoma (7, 8). Wang et al. (7) treated U2OS cells (osteosarcoma cells) with low concentrations of anlotinib and cisplatin (CDDP) to detect the effects of both cell proliferation and apoptosis, and the results showed that anlotinib could inhibit the proliferation of osteosarcoma cells and promote CDDP-induced apoptosis. Anlotinib combined with chemotherapy is better than chemotherapy alone in the remission of disease, anlotinib can enhance the sensitivity of tumor chemotherapy, and the two have a synergistic effect. A retrospective study found that radiotherapy can promote exposure to tumor tissue antigens, and the combination with anlotinib can enhance the anlotinib-induced immune response (9).

We herein report a case of recurrent pelvic osteosarcoma successfully treated with anlotinib combined with chemotherapy and radiotherapy. Under multimodal combination therapy, the patient achieved successful limb salvage after relapse and was alive and disease-free at 25 months. Thankfully, the patient has not developed lung metastases to date. The patient consented to the publication of the data concerning the case.

## Case report

**Figure 1:**
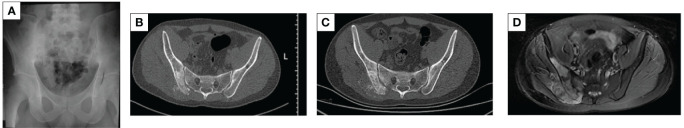
X-ray, CT, and MRI images. **(A)** Osteoblastic destruction of the right sacroiliac joint with a periosteal reaction. **(B)** The local discontinuity in the bone cortex of the right sacroiliac joint before chemotherapy, along with osteogenic bone destruction, large surrounding soft tissue mass, and unclear boundaries. **(C)** The bone image of multiple lamellar tumors in the soft tissues after neoadjuvant chemotherapy, with obvious calcification. **(D)** An MRI after neoadjuvant chemotherapy revealed a small local soft tissue mass and reduced peripheral inflammatory response.

**Figure 2:**
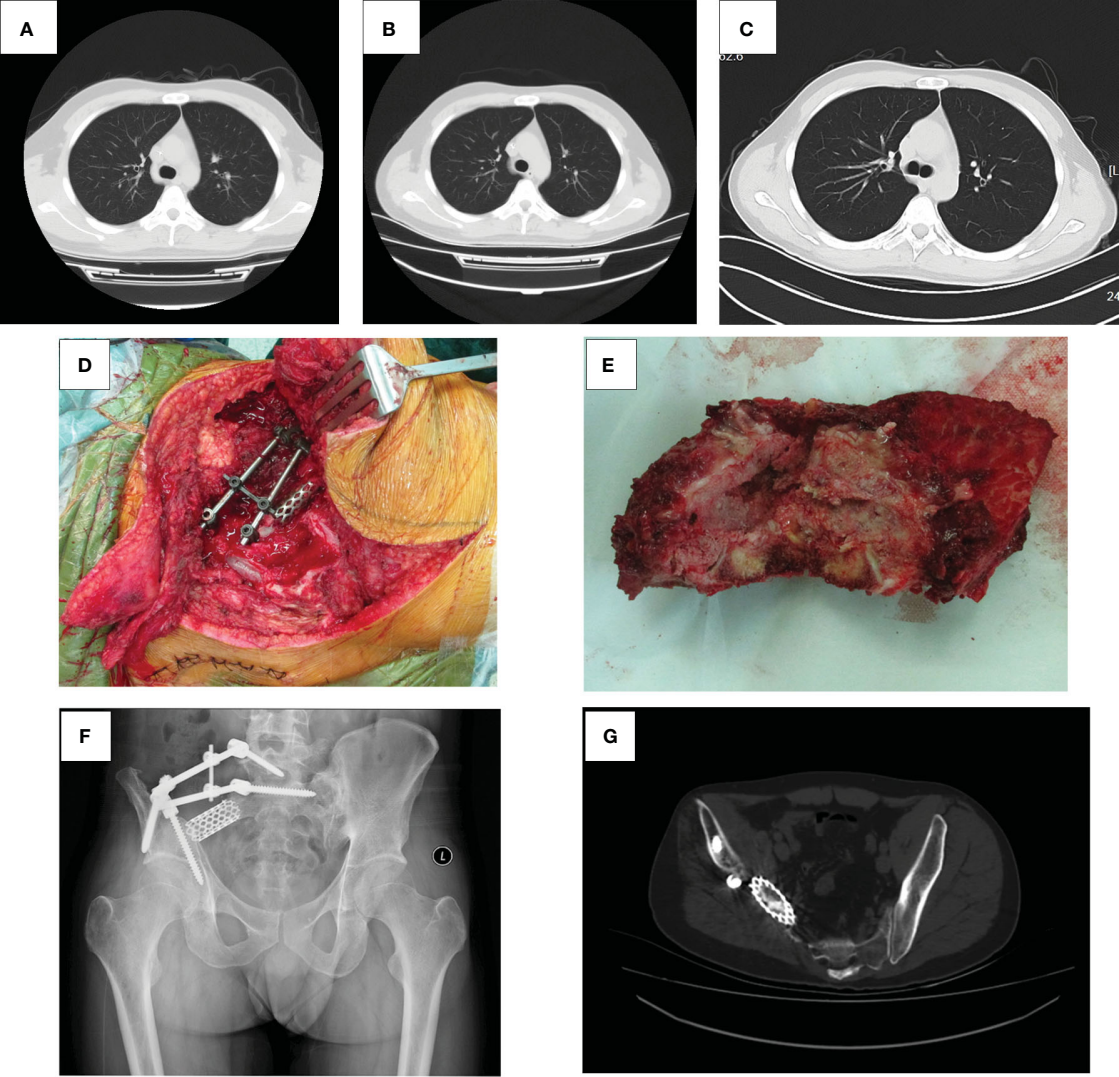
Lung CT, operative photograph, tumor specimen, X-ray, and pelvic CT after surgery. **(A)** The lung CT scan results of the patient at the first admission. **(B)** The lung CT plain scan results of patients admitted for chemotherapy in sequence. **(C)** The lung CT plain scan results of the patient on September 5, 2023. Lung CT showed no abnormal density in both lungs. **(D)** The tumor was completely resected, pedicle and iliac nails were inserted, and titanium cages were placed. **(E)** Completely resected tumor tissue. **(F)** Partial bone loss in the right ilium and sacrum, with metal fixation in the corresponding areas. **(G)** Partial absence of the right sacral wing, ilium, gluteus, and iliac muscles on pelvic CT, with metal fixation shadows visible in the right sacral ilium and numerous radiating artifacts around them.

**Figure 3:**
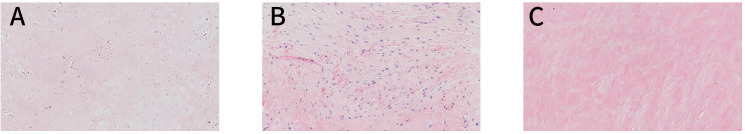
Pathology images. **(A)** A pathological image captured with an optical microscope at ×200 magnification and an image in which some heterotypic cell components were detected in the tissues, some cells showed abundant cytoplasm, and some areas were necrotic; these observations are consistent with the chondroblast-type osteosarcoma. **(B)** A pathology image of a post-recurrence puncture captured with an optical microscope at ×200 magnification and an image in which the chondrosarcomatoid components were detected. **(C)** A pathological image showing the puncture at the end of multimodal combination therapy, as captured with an optical microscope at ×200 magnification and an image in which the tissue submitted for examination was striated muscle-grade fibrous adipose tissues, with no tumor cells detected.

A 29-year-old male patient was hospitalized locally 5 years ago for low back pain. The pelvic radiograph showed osteoblastic destruction of the right sacroiliac joint with periosteal reaction and a high probability of malignant bone tumor (**Figure 1A**). A computed tomography (CT) scan revealed an irregular bone destruction area in the right sacroiliac joint and a large soft tissue mass with unclear boundaries (**Figure 1B**). Lung CT displayed no significant visceral metastasis (**Figure 2A**). Puncture pathology indicated osteosarcoma. The patient visited our hospital for further diagnosis and treatment. Overcoming chemotherapy taboos, neoadjuvant DIA (cisplatin 120 mg/m^2^ for 1 day, epirubicin 40 mg/m^2^ for 3 days, and ifosfamide 2 g/m^2^ for 5 days) chemotherapy regimens were initiated once every 2 weeks for two times. CT repeated after chemotherapy revealed patellar high and low mixed density in the right iliac bone, mass formation of surrounding soft tissues, multiple lamellar tumor bones in the soft tissue, and obvious calcification (**Figure 1C**). Magnetic resonance imaging (MRI) exhibited a small local soft tissue mass and reduced surrounding inflammatory responses (**Figure 1D**). Accordingly, surgery was planned for the patient. On November 14, 2018, tumor resection and reconstruction with screw rod fixation were performed (**Figures 2D, E**). Postoperative pathology confirmed that the patient had chondrocyte osteosarcoma (**Figure 3A**), and the tumor necrosis rate was approximately 90%, consistent with the post-chemotherapy response and Huvos rating system Grade III. Postoperative chemotherapy was performed for nine cycles (the dose and regimen of chemotherapeutic drugs were the same as those before the operation). Local plain radiography and CT were conducted regularly during chemotherapy (**Figures 2F, G**), and no recurrence or distant metastasis was noted (**Figure 2B**).

**Figure 4:**
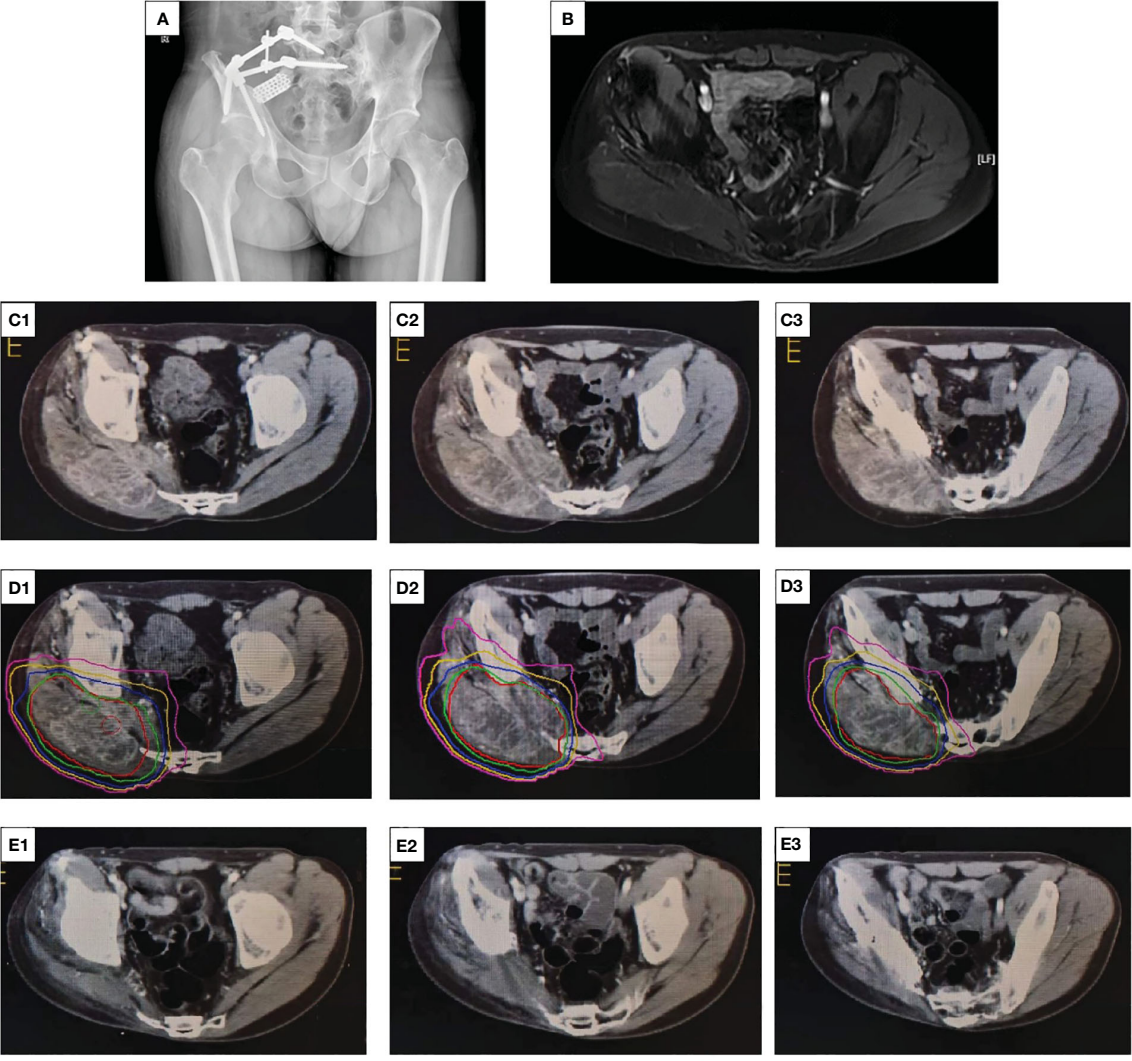
X-ray, contrast-enhanced CT, and radiation dose segmentation diagram and MRI images. **(A)** An abnormally low-density shadow within the right gluteus maximus with an unclear boundary. **(B)** Irregular T2 signals in the right residual muscle with unclear boundary. **(C1, 2, 3)** The enhanced CT scan of the patient’s pelvis before radiotherapy, with the red arrow indicating the mass. **(D1, 2, 3)** The dose segmentation of radiotherapy. **(E1, 2, 3)** The enhanced CT of the pelvis at the end of multimodal therapy, indicating no tumor tissues.

Unfortunately, on March 18, 2021, the patient developed sudden mild pain at the surgical site. A palpable mass was noted around the original surgical incision. Therefore, the 10th course of adjuvant postoperative chemotherapy was suspended. X-ray showed an abnormally low-density shadow within the right gluteus maximus with an unclear boundary (**Figure 4A**). Enhanced CT revealed that the soft tissue tumor was large in volume, with unclear boundaries, was of low density, and exhibited uneven edge enhancement and abundant internal blood flow (**Figure 4C**). MRI showed irregular signals in the right residual muscle with a maximum cross-sectional area of 3.8 × 2.2 cm, and the boundary was not clear (**Figure 4B**). To confirm the diagnosis, a B-ultrasound-guided needle biopsy was performed (**Figure 3B**), which indicated local recurrence. Following recurrence, if the tumor is large and is located close to abdominal organs, R0 resection cannot be performed with the limb preserved.

After this challenge was communicated to the patient and his family, the patient refused surgery and preferred to continue conservative treatment. Multidisciplinary consultation recommended 12 mg once daily anlotinib on a 2-week on and 1-week off basis. Combined with intensity-modulated radiotherapy (IMRT), a total dose of 60 Gy equivalents (300 cGy/time, 20 times in total) was administered over 4 weeks, which resulted in local control (**Figure 4D**). In the treatment course, we never disregarded the significance of chemotherapy in recurrent osteosarcoma and continued to administer six courses of adjuvant chemotherapy and dose enhancement. The first one to four courses included a combination of DDP and EPI. EPI (50 mg/m^2^) was dissolved in an equal volume of 5% glucose and was administered through an intravenous infusion (i.v. drip) on day 1 as previously described. DDP was dissolved in saline and administered through an intravenous infusion (i.v. drip) at 140 mg/m^2^ for 1 day, with adequate hydration and antiemesis (HT3 receptor blockers, such as ondansetron, were used as antiemetics) treatment. Because the cumulative dose of EPI in the patient was reaching the critical value of 900–1,000 mg/m^2^, the DDP + IFO regimen was adopted for the last two chemotherapy courses. The DDP dose and administration method remained unchanged, and 2 g/m^2^ IFO was dissolved in saline and administered for 5 days.

After the multimodal combination therapy, the tumor was evaluated by imaging and through cytology. Contrast-enhanced CT revealed no obvious soft tissue mass (**Figure 4E**). On May 16, 2023, the patient was rehospitalized, and a puncture biopsy was performed at the primary site. Pathological examination revealed both the striated muscle and fibroadipose tissue (**Figure 3C**). On this admission, the patient presented hyposensitivity on his right foot skin. The strength of the right dorsal extensor and tibial anterior muscles was both grade 0, which resulted in common peroneal nerve damage after radiotherapy. The current functional outcome, as measured by the functional rating system of the Musculoskeletal Tumor Society (MSTS), was 19. The patient was diagnosed with osteosarcoma in 2018, but no signs of lung metastasis were observed (**Figure 2C**).

## Discussion

Pelvic osteosarcoma presents a substantial therapeutic challenge because of the potential late symptom onset, metastatic dissemination at diagnosis, and inherent difficulties of wide surgical resection secondary to the complex and critical pelvic anatomy. The estimated 5-year survival rate associated with pelvic osteosarcoma is 18%–38%, and resection is frequently associated with severe functional loss. Tumors located near the sacroiliac joint or in the ilium are usually larger and have a higher pulmonary metastasis rate and a lower operative treatment rate than those in other locations. The sacrum is considered to be mostly prone to recurrence (4, 10). Limb preservation has recently become more popular than conventional hemipelvectomy. Patients who continue to undergo hindquarter amputation may have more advanced disease, for example, with the involvement of great vessels or a major nerve (11). Therefore, conservative radiotherapy/chemotherapy and targeted drugs are being attempted for patients with recurrent pelvic osteosarcoma to achieve a higher clinical outcome (1, 3, 11).

In the present case, three treatment modalities were adopted to maximize the treatment effect. A standard rescue plan for recurrent or refractory osteosarcoma is lacking. First, we continue to use the combination of DDP, EPI, and IFO first-line chemotherapeutic agents at increased doses as the conventional chemotherapy regimen for recurrent pelvic osteosarcoma. Although second-line chemotherapy drugs such as topotecan, irinotecan, imatinib mesylate, and temozolomide are emerging, and patients can tolerate them despite their toxicities and side effects, the associated effective rates are low, and the survival times are short. The 2-year overall survival (OS) and progression-free survival (PFS) rates of the vincristine, irinotecan, and temozolomide combination, used as a salvage regimen for recurrent or refractory sarcomas in children and young adults, were 45.5% and 25.4%, respectively. Moreover, long-term survival results remain unsatisfactory (12). By contrast, some first-line drug combinations have shown clinical activity (13–15).

Second, local control is the major issue in pelvic osteosarcoma treatment. It has been reported that the 10-year local control rate, disease-free survival rate, and overall survival rate of patients receiving adjuvant radiotherapy after neoadjuvant chemotherapy were 82%, 78%, and 53%, respectively (16). The National Comprehensive Cancer Network (NCCN) guidelines for osteosarcoma point out that osteosarcoma is not sensitive to radiation therapy, the effect of radiation therapy alone is poor, and it can be used as a means of comprehensive treatment for osteosarcoma in areas that are not or difficult to resect (such as the sacrum, pelvis, and spine) (17). IMRT is administered in conjunction with chemotherapy because chemotherapeutic agents act as radiosensitizers when combined with radiotherapy during administration. Patients with locally diseased osteosarcoma who receive a combination of radiotherapy and chemotherapy have a 5-year mean OS of 61%–75% (18). Radiotherapy may improve the outcome in patients where achieving adequate surgical margins is not possible (11). According to THOMAS F et al. (19), highly conformal radiotherapy techniques, such as IMRT and/or proton beam radiotherapy, play a crucial role in improving local tumor control rates for the skull, head, neck, spine, and pelvis. These techniques can improve the delivery of radiation to the target volume while minimizing scattering to surrounding organs (20). For definitive radiation therapy, 55–60 Gy doses are administered with a conventional daily fractionation of 1.8 Gy (21).

Multitarget tyrosine kinase inhibitors (TKIs) exhibit antitumor activities against osteosarcoma. The National Comprehensive Cancer Network guidelines have recommended TKIs as second-line therapy for advanced osteosarcoma that progresses after chemotherapy (17). The postoperative pathology of our patient was chondroblastic osteosarcoma with low chemosensitivity. Therefore, antiangiogenic drugs and chemotherapy were used simultaneously, which plays a role in therapeutic sensitization. Anlotinib inhibited migration and invasion of osteosarcoma cells by suppressing MET and VEGFR2 phosphorylation and activation of the downstream signaling pathway. In phase II trials involving previously heavily treated advanced osteosarcoma patients, anlotinib achieved a 3-month PFS rate of 75.86% and a median PFS of 4.8 months. In a 143B-Luc orthotopic osteosarcoma model, anlotinib significantly inhibited the growth of transplanted tumor cells and lung metastasis (22). Compared with other receptor tyrosine kinase (RTK) inhibitors, anlotinib is absorbed quickly, has a longer half-life, inhibits more targets, and has fewer and milder side effects, especially compared to the thrombocytopenia and neutropenia side effects of sunitinib (23). Therefore, after relapse, anlotinib was initiated as a sequential treatment following chemotherapy in this study. This is because, in the absence of targeted therapy, chemotherapy alone has produced rather discouraging clinical outcomes in osteosarcoma patients with refractory or metastatic disease recurrence, with 4-month PFS of as low as 12% (24, 25). Although a single application of antiangiogenic drugs can significantly improve PFS, the ORR of anlotinib is only 6.89%. In summary, combined therapy is required in clinical applications.

Targeted therapy and chemotherapy will inevitably cause different degrees of adverse reactions. The study results revealed that the combination of chemotherapy and anlotinib can largely benefit patients with advanced/metastatic soft tissue sarcoma (STS) in terms of survival, along with good tolerance. The most common grade 3 and 4 adverse events are febrile neutropenia (9%), leukopenia (19%), thrombocytopenia (3%), anemia (6%), anorexia (6%), vomiting (3%), and hypertension (6%) (22). Our patient developed grade 3 myelosuppression during treatment and was successfully treated with a granulocyte-stimulating factor. Subsequently, he did not develop more significant toxicity. In total, six cycles of combination therapy were tolerated. His thyroid, liver, and kidney functions were all within the normal range.

Because different drugs may interact through multiple mechanisms, combination therapy is highly complex. Methods to apply tumor combination therapy more reasonably, more effectively, and with less toxicity need to be further explored. The effect of anlotinib combined with adjuvant chemotherapy and radiotherapy on patients with recurrent pelvic osteosarcoma requires to be confirmed in numerous subsequent clinical trials.

## Data availability statement

The original contributions presented in the study are included in the article/supplementary material. Further inquiries can be directed to the corresponding author.

## Ethics statement

The studies involving humans were approved by The 960th Hospital Ethics Department of the Chinese People’s Liberation Army Joint Logistic Support Force. The studies were conducted in accordance with the local legislation and institutional requirements. The participants provided their written informed consent to participate in this study. Written informed consent was obtained from the individual(s) for the publication of any potentially identifiable images or data included in this article.

## Author contributions

QC: Resources, Writing – original draft. KZ: Supervision, Validation, Writing – review & editing. MX: Supervision, Writing – review & editing. GH: Resources, Writing – review & editing. NY: Resources, Writing – review & editing. YX: Resources, Writing – review & editing.
